# Thiopurine dose intensity and treatment outcome in childhood lymphoblastic leukaemia: the influence of thiopurine methyltransferase pharmacogenetics

**DOI:** 10.1111/bjh.13240

**Published:** 2014-11-29

**Authors:** Lynne Lennard, Cher S. Cartwright, Rachel Wade, Ajay Vora

**Affiliations:** ^1^ Academic Unit of Clinical PharmacologyUniversity of SheffieldSheffieldUK; ^2^ Clinical Trials Service UnitOxfordUK; ^3^ Department of Paediatric HaematologyChildren's HospitalSheffieldUK

**Keywords:** thiopurine methyltransferase, mercaptopurine, thioguanine, cytopenias, acute lymphoblastic leukaemia

## Abstract

The impact of thiopurine methyltransferase (*TPMT*) genotype on thiopurine dose intensity, myelosuppression and treatment outcome was investigated in the United Kingdom childhood acute lymphoblastic leukaemia (ALL) trial ALL97. *TPMT* heterozygotes had significantly more frequent cytopenias and therefore required dose adjustments below target levels significantly more often than *TPMT* wild‐type patients although the average dose range was similar for both genotypes. Event‐free survival (EFS) for patients heterozygous for the more common TPMT*1/*3A variant allele (*n* = 99, 5‐year EFS 88%) was better than for both wild‐type TPMT*1/*1 (*n* = 1206, EFS 80%, *P *= 0·05) and TPMT*1/*3C patients (*n* = 17, EFS 53%, *P *= 0·002); outcomes supported by a multivariate Cox regression analysis. Poor compliance without subsequent clinician intervention was associated with a worse EFS (*P *= 0·02) and such non‐compliance may have contributed to the poorer outcome for TPMT*1/*3C patients. Patients prescribed escalated doses had a worse EFS (*P *= 0·04), but there was no difference in EFS by dose intensity or duration of cytopenias. In contrast to reports from some USA and Nordic trials, *TPMT* heterozygosity was not associated with a higher rate of second cancers. In conclusion, TPMT*1/*3A heterozygotes had a better EFS than *TPMT* wild‐type patients. Thiopurine induced cytopenias were not detrimental to treatment outcome.

Mercaptopurine and thioguanine (2‐amino 6‐mercaptopurine, tioguanine) are purine analogue drugs that have been used as cytotoxic agents in the treatment of leukaemias for over 50 years (Burchenal *et al*, 1953; Murphy *et al*, 1955). Both these thiopurine drugs are metabolized to form thioguanine nucleotide metabolites (TGNs), which have cytotoxic and immunosuppressive properties. Incorporation of TGN into DNA initiates subsequent cytotoxicity (Tidd & Paterson, 1974; Warren *et al*, 1995; Karran, 2006) and TGN inhibition of intracellular signalling pathways induces apoptotic cell death whilst one TGN, thioguanine triphosphate, suppresses lymphocyte activation by binding to RAC1 in place of endogenous guanine triphosphate (Tiede *et al*, 2003; Poppe *et al*, 2006; Marinkovic *et al*, 2014).

TGN production from the inactive thiopurine drug is regulated by the polymorphic enzyme thiopurine methyltransferase (TPMT). Thioguanine forms TGNs directly but mercaptopurine forms additional intermediate nucleotide metabolites; one of these, mercaptopurine nucleotide (mercaptopurine riboside 5′‐monophosphate or 6‐thioinosinic acid) is a good substrate for TPMT, and the resulting methyl‐mercaptopurine nucleotide metabolites (MeMPNs) are formed at the expense of TGNs. TPMT deficiency (1 in 300 individuals) is associated with bone marrow failure when such patients are treated with standard doses of thiopurine drugs (Lennard *et al*, 1989; Evans *et al*, 1991; McBride *et al*, 2000); a less severe myelosuppression can develop in *TPMT* heterozygotes (‘intermediate’ activity, 11% of subjects), (Weinshilboum & Sladek, 1980; Lennard *et al*, 1990; Relling *et al*, 1999a).

For thiopurine drugs the therapeutic window between toxicity and efficacy is narrow. For children with acute lymphoblastic leukaemia (ALL) treated with mercaptopurine, those individuals with lower TPMT activities and/or higher TGN levels have a lower relapse‐risk (Lennard & Lilleyman, 1989; Schmeigelow *et al*, 1995; Relling *et al*, 1999a). Traditionally, daily oral mercaptopurine is taken during the long‐term maintenance phase of chemotherapy in childhood ALL. Within the United Kingdom (UK) ALL protocols this phase of treatment lasts for 2 to 3 years and maintains the disease remission that has been induced by other potent cancer chemotherapeutic agents; the central role of mercaptopurine in maintaining disease remission is illustrated by the worse outcome observed in all attempts to shorten the duration of mercaptopurine‐containing maintenance chemotherapy such that the duration of total therapy falls below 2 years (Schrappe *et al*, 2000; Richards *et al*, 1996).

In the UK Medical Research Council national ALL trial ALL97, children and young adults were randomized to receive either mercaptopurine or thioguanine in the maintenance phase. The clinical results of the ALL97 thiopurine randomization have been previously reported and showed no difference in efficacy between thioguanine and mercaptopurine. A lower risk of central nervous system (CNS) relapse for thioguanine was offset by an increased risk of death in remission, (mainly due to infections), and an increased risk of thioguanine‐induced veno‐occlusive disease (VOD) (Lennard *et al*, 2006; Vora *et al*, 2006). We have previously reported the TPMT genotype‐phenotype discordance and the superiority of *TPMT* genotyping for the detection of the *TPMT* heterozygote in this patient cohort, together with the influence of *TPMT* genotype on thiopurine metabolite accumulation (Lennard *et al*, 2013). In this paper we report on the influence of the *TPMT* genetic polymorphism on thiopurine dose intensity and myelosuppression, and the resulting impact on treatment outcome.

## Methods

### Patients and chemotherapy

ALL97 [International Standard Randomized Controlled Trial Number (ISRCTN) registration number ISRCTN26727615] was a randomized comparison of dexamethasone versus prednisone and mercaptopurine versus thioguanine in patients aged 1 to 18 years. The patient cohort has been previously described (Vora *et al*, 2006). Treatment centres obtained local ethics committee approval and informed consent from patients and/or parents before entering children into the trial. The trial had an add‐on thiopurine biological study. ALL97 was modified in November 1999 and termed ALL97/99, but the randomizations and biological studies were retained, as was the registered ALL97 trial name. Details of the treatment regimens and modifications have been previously reported (Mitchell *et al*, 2005, 2009; Vora *et al*, 2006). ALL97 closed to accrual in June 2002. At closure, all of the children randomized to thioguanine who had not finished maintenance chemotherapy were transferred to mercaptopurine.

During maintenance patients received daily oral randomized thiopurine, weekly methotrexate, monthly intravenous vincristine and 5 days of randomized steroid. Maintenance was given for 2 years in ALL97 but was increased to 3 years, for boys only, in ALL97/99. When ALL97 was superseded by ALL97/99, all boys had maintenance increased to 3 years; effectively only boys diagnosed in 1997 received 2 years maintenance. The thiopurine dose was titrated to toxicity from a standard protocol dose (thioguanine 40 mg/m^2^ and mercaptopurine 75 mg/m^2^; 100% protocol dose) for both *TPMT* heterozygous and wild‐type patients. Patients with TPMT deficiency (homozygous for two variant low activity alleles) were titrated from a starting dose of 10% protocol dose (7·5 mg/m^2^ mercaptopurine, 4·0 mg/m^2^ thioguanine). The dose titration protocols for the ALL97 and ALL97/99 phases of the trial have been described elsewhere (Vora *et al*, 2006). Briefly, ALL97 had a more aggressive titration protocol, with dose adjustments every 4 weeks if the neutrophil counts remained above 1·0 × 10^9^/l and platelet counts above 100 × 10^9^/l, whilst ALL97/99 dose adjustments occurred at the start of each 12‐week maintenance cycle to maintain the neutrophil count between 0·75 and 1·5 × 10^9^/l and platelet count over 75 × 10^9^/l. For both ALL97 and ALL97/99, the thiopurine dose was reduced if the neutrophil count fell below 0·75 × 10^9^/l (or platelet count 75 × 10^9^/l) and withdrawn if neutrophil counts fell <0·5 × 10^9^/l (or platelet counts <50 × 10^9^/l).

### Thiopurine dosage calculations

Throughout treatment, drug dosage was recorded weekly and cell counts were recorded at monthly or fortnightly intervals (or more frequently) as appropriate. These hand‐written forms were forwarded and collated by the Clinical Trials Service Unit, Oxford. Databases were designed to capture the thiopurine dosage and cell count information. The database started at Week 8, after the induction and consolidation blocks. High‐risk patients (ALL97 protocol HR1 and ALL97/99 regimen C, which contained additional multi‐drug chemotherapy during the first year) were excluded from the thiopurine dosage analysis, as were children who relapsed or died during the first year of chemotherapy.

Patients included in the thiopurine dosage and cell count analysis had achieved remission by the end of induction and had completed forms detailing at least 95% of maintenance treatment received from Week 8. The total number of weeks that thiopurine could have been prescribed during maintenance cycles was calculated. Some children required a period of cell count recovery following the delayed intensive blocks given during year 1, at the start of a maintenance phase of thiopurine treatment. This time was calculated and subtracted from the total number of weeks that thiopurine could have been prescribed as these cytopenias were influenced by other chemotherapy. The daily thiopurine dose (mg/m^2^) was totalled and the number of weeks that each child was prescribed the standard protocol dose (100% dose = 75 mg/m^2^ mercaptopurine or 40 mg/m^2^ thioguanine), escalated doses (>100%), reduced doses, or that thiopurine was withdrawn, was calculated. To calculate the average daily thiopurine dose, the totalled daily dose (per m^2^) throughout maintenance was divided by the time (days) that thiopurine could have been prescribed. To enable comparisons between thiopurines and the use of dosage data from those children who switched from thioguanine to mercaptopurine during treatment, the percentage (%) standard protocol dose for each thiopurine was calculated. The % time when thiopurines were withdrawn, or the dose reduced or escalated, was also calculated, as was the % time with neutropenia (neutrophil count <1·0 × 10^9^/l and <0·5 × 10^9^/l) and thrombocytopenia (platelet count <100 × 10^9^/l).

### Blood samples

*TPMT* genotype was determined in a diagnostic lithium heparin blood sample (5 ml), and/or a blood sample taken during remission maintenance chemotherapy. The blood sample protocol has been previously described (Lennard *et al*, 2013). TPMT activity was also measured in these blood samples; values were previously reported by Lennard *et al* (2013). Clinicians were informed if the patient had TPMT activity (the thiopurine dose to be adjusted, based on cell counts, from the protocol standard dose) or if the patient was TPMT‐deficient (start the thiopurine dose at 10% of the protocol standard dose, adjust on the basis of cell counts). During remission maintenance chemotherapy blood samples were requested at the earliest point in interim maintenance when patients were tolerating thiopurines at the standard protocol, or the maximum tolerated dose; thiopurine metabolites measured in this sample served as a reference value. Within the thiopurine study of the ALL97 trials, additional blood samples were forwarded from clinicians on an *ad hoc* basis when patients were either unduly sensitive to thiopurines, or tolerating high doses (Lennard *et al*, 2013). Thiopurine metabolite values were fed back to the clinician, as a check on compliance with oral chemotherapy, prior to dose escalation.

### Thiopurine assays

Thiopurine metabolite concentrations were measured by high performance liquid chromatography (HPLC); the lower limit of detection for TGNs and MeMPNs was 6 and 15 pmol/8 × 10^8^ red cells, respectively (Lennard & Singleton, 1992; Lennard *et al*, 2013). Blood samples were genotyped for TPMT*3A, TPMT*3B and TPMT*3C, by amplification of exons 7 and 10 of the *TPMT* gene (TPMT*3A is an exon 7 and 10 double mutant) as previously described (Lennard *et al*, 2013). TPMT *2 was detected by sequencing exon 5 of the *TPMT* gene and novel sequence variations were identified by sequencing the *TPMT* open reading frame from exon 3 to exon 10 as previously described (Otterness *et al*, 1997; Lennard *et al*, 2013).

### Compliance

Non‐compliance with oral chemotherapy was suspected when patients maintained high cell counts despite tolerating long‐term thiopurines at ≥100% doses (Lennard *et al*, 1995). With respect to mercaptopurine, low concentrations of both TGN and MeMPN metabolites (both metabolites < lower quartile concentrations) have been used as an index of partial compliance; these metabolites are products of competing pathways and show an inverse correlation (Lennard *et al*, 1995). Higher TPMT activity is associated with lower TGN concentrations and higher MeMPNs whilst lower TPMT activity is associated with higher TGNs and lower MeMPNs. Overt mercaptopurine non‐compliance was defined as both TGN and MeMPN metabolites at or below the lower limit of detection. For thioguanine, due to the rapid accumulation of thioguanine‐derived TGNs in the red cell following an oral dose, non‐compliance was suspected if metabolite concentrations were <750 pmol TGNs (Lennard *et al*, 2013).

### Statistical analysis

The Anderson‐Darling test was used to examine the fit of observations to a normal distribution. Differences between groups were compared by the Chi‐square statistic, or the Mann‐Whitney test; quartile analysis of the equality of medians was by the Kruskal‐Wallis test, quartile analysis of survival was by the log‐rank test for trend. Outcome analysis was of event‐free survival (EFS), with an event defined as time to relapse or death, as used in previous ALL97 analysis (Vora *et al*, 2006). Kaplan‐Meier curves were calculated and comparisons between groups were performed by the log‐rank statistic with stratification by age, gender, white blood cell (WBC) count at diagnosis, trial phase (ALL97 or ALL97/99) and steroid received (prednisone, dexamethasone). Overall EFS (from randomization at the start of treatment) was carried out for any analysis involving all patients. For the outcome analysis of dosing and cell count data, patients joined the analysis at the end of year one (i.e., patients with events in year 1 were excluded) and EFS was calculated from this starting point (termed jEFS), stratified by length of maintenance (two or three years). All *P* values are two‐sided, a *P* value <0·05 was considered statistically significant. Cox regression multivariate analysis was used to test whether the effects of variables were independent. Analyses were to the annual follow up of 30th April 2011; median follow‐up for survivors 11·3 years (range 9·6 to 14·3 years). Statistical analyses were by SAS (version 9.2; SAS, Cary, NC, USA) or Minitab 16.

## Results

### Patient numbers

**Figure 1:**
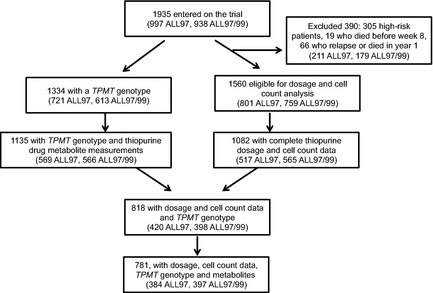
Trial data profile. The numbers of individual patients providing blood samples for thiopurine analysis by trial phase, ALL97 and ALL97/99. *TPMT*, thiopurine methyltransferase.

The patient numbers and data available are summarized in Fig 1. There was no difference with respect to gender, age, WBC count at diagnosis, steroid randomization or thiopurine randomization between patients with and without thiopurine data.

Thiopurine metabolites and TPMT data have been previously reported for the thioguanine versus mercaptopurine randomized cohort in the ALL97 trial (Lennard *et al*, 2013). This current paper contains an additional 14 children, who were recruited whilst the trial was still open but the mercaptopurine versus thioguanine randomization was closed, who received non‐randomized mercaptopurine.

### TPMT genotype and thiopurine metabolites

*TPMT* genotype was available for 1334 patients (69% of patients entered onto ALL97); 1160 were white and 174 belonged to other ethnic groups (71 Asian (Indian sub‐continent), 44 mixed race, 20 black, 6 Oriental and 33 unknown or non‐Caucasian). 1206 patients were homozygous wild‐type TPMT*1/*1 and 128 patients had low activity variant alleles (99 TPMT*1/*3A; 17 TPMT*1/*3C; 4 TPMT*1/*2; two children with the rare alleles TPMT*1/*9, TPMT*1/*21; three children with novel alleles TPMT*1/*32, TPMT*1/*33, TPMT*1/*34; one compound heterozygote TPMT*2/*3A; one homozygous TPMT*3A/*3A and one TPMT*3C/*3C). The TPMT*3B allele was not detected.

Thiopurine metabolite assays were available for 1135 of the 1334 *TPMT* genotype children, 426 randomized to thioguanine and 709 taking mercaptopurine. The metabolite concentrations differ by genotype (Lennard *et al*, 2013) and are summarized in Table 1: there was no heterogeneity with respect to gender. Very low metabolite concentrations when supposedly taking high doses thiopurines was a strong indication that non‐compliance with oral chemotherapy was a problem in all *TPMT* groups. Comparisons between *TPMT* heterozygotes were not possible with thioguanine (only four TPMT*1/*3C children), but within the mercaptopurine cohort TPMT *1/*3C children (*n *= 12) had significantly lower TGN concentrations than TPMT *1/*3A children (*n *= 53) despite having similar drug dosages and TPMT activities (Lennard *et al*, 2013); TPMT *1/*3C median TGNs 608 pmol/8 × 10^8^ red cells (range 288 to 910) and TPMT *1/*3A median TGNs 802 (range 132 to 2228), median difference 192 pmol (95% confidence interval [CI] 10 to 425), *P *= 0·05). MeMPN concentrations were also lower in TPMT *1/*3C patients; TPMT *1/*3C median MeMPN 2061 pmol/8 × 10^8^ red cells (range 60 to 10746) and TPMT *1/*3A median MeMPN 4542 (range 84 to 38 386), median difference 2190 pmol (95% CI −54 to 5180), *P *= 0·06).

**Table 1:** Thiopurine methyltransferase genotype and metabolite formation

	Wild‐type TPMT*1/*1	Heterozygous *TPMT*	Median difference (95%CI)
TG patients	385	40	
TG dose, mg/m^2^	40 (14–78)	40 (18–63)	−1·0 (−2·0 to −0·001), *P *= 0·0525, NS
TG‐TGNs pmol	1904 (36–4336)	2468 (174–6730)	504 (206 to 802), *P *= 0·0009
MP patients	636	71	
MP dose mg/m^2^	75 (6–125)	74 (14–113)	−1·0 (−4·0 to 0·000), *P *= 0·06, NS
MP‐TGNs pmol	360 (0–1216)	754 (132–2228)	394 (326 to 466), *P *< 0·0001
MP‐MeMPNs pmol	10702 (0–141772)	4078 (60–38386)	−5464 (−7278 to −3808), *P *< 0·0001

The thiopurine dose is that tolerated at the time of metabolite measurement. The standard protocol dose was 75 mg/m^2^ mercaptopurine and 40 mg/m^2^ thioguanine. Thiopurine metabolite formation differs by genotype, there was no heterogeneity by gender. TPMT, thiopurine methyltransferase; TG, thioguanine cohort; MP, mercaptopurine cohort; TGNs, thioguanine nucleotides; MeMPNs, methylmercaptopurine nucleotides; NS = not significant. TGN and MeMPN units are pmol/8 × 10^8^ red cells. Values are given as median (range). The *TPMT* variant alleles quantified were TPMT *1/*2 (MP *n *= 3; TG *n *= 1), TPMT *1/*3A (MP *n *= 53; TG *n *= 33), TPMT *1/*3C (MP *n *= 12; TG *n *= 4), TPMT *1/*9 (MP *n *= 1) and TPMT *1/*21 (TG *n *= 1) and the novel alleles TPMT *1/*32 TPMT *1/*33 TPMT *1/*34 (*n *= 1 each taking MP, MP and TG respectively). The above analysis excludes 3 TPMT‐deficient patients. The TPMT*3A/*3A and TPMT *2/*3A patients have been previously reported (Lennard *et al*, 2013), the TPMT*3A/*3A child eventually settled on 2·5 mg/m^2^ per day thioguanine and the TPMT *2/*3A child 7·5 mg/m^2^ mercaptopurine on alternate days; TGNs were 2252 pmol and 1670 pmol, respectively. The TPMT *3C/*3C child had 1784 pmol TGNs at 15 mg/m^2^ mercaptopurine.

### Thiopurine dosage and cell counts

The average daily dose was 77% of the standard protocol dose and this did not differ between the ALL97 and ALL97/99 phases of the trial (Table 2). As expected in a protocol in which the drug dosage was adjusted based on cell counts, quartile analysis of average % daily dose showed that those patients with higher doses had significantly higher cell counts; from the lower to the higher quartile (Q) of average % dose (Q1 = 3·0 to 65·2%; Q2 = 65·3 to 76·7%; Q3 = 76·8 to 86·8%; Q4 = 86·9 to 162·4%) the median % weeks with a neutrophil count <1·0 × 10^9^/l was 32, 28, 23 and 15, respectively (*P *< 0·001), the median % weeks with a neutrophil count <0·5 × 10^9^/l was 14·3, 12·5, 9·0 and 5·9, respectively (*P *< 0·001) and the median % weeks with thrombocytopenia was 10·0, 4·9, 2·3, 2·1, respectively (*P *< 0·001). There was no difference in the % time with neutropenia between the ALL97 and ALL97/99 phases, but the incidence of thrombocytopenia was greater in ALL97 (Table 2). Throughout both trial phases, thioguanine caused more thrombocytopenia than mercaptopurine but the degree of thrombocytopenia was greater in ALL97 than ALL97/99 (Table 3). Although thioguanine‐related VOD and the resulting endotheliitis may partly account for the excess thioguanine‐related risk of thrombocytopenia, the additional risk in the ALL97 phase is unlikely to be related to VOD as it was more prevalent in the ALL97/99 phase than in ALL97 (Lennard *et al*, 2006; Vora *et al*, 2006). The lower platelet counts were more evident in older children (*P *< 0·0001, Table 2) and was seen in both phases of the trial.

**Table 2:** Thiopurine dosage and myelosuppression according to clinical features

Subgroup	n	Average dose (% of protocol)	*P*	% time at no dose	*P*	% time dose escalated	*P*	% time with neutropenia (<0·5 × 10^9^/l)	*P*	% time with thrombocytopenia (<100 × 10^9^/l)	*P*
Trial											
ALL97	517	76·6 (24·2–134·2)	0·08	16·8 (0·0–62·2)	<0·0001	7·2 (0·0–72·3)	<0·0001	23·9 (2·4–57·4)	0·8	4·9 (0·0–63·3)	<0·0001
ALL97/99	565	76·7 (3·0–162·4)		14·4 (0·0–97·0)		3·0 (0·0–84·2)		23·6 (0·8–62·9)		2·4 (0·0–81·9)	
Sex											
Male	576	79·6 (3·0–162·4)	<0·0001	13·8 (0·0–97·0)	<0·0001	7·0 (0·0–84·2)	<0·0001	22·0 (0·8–62·9)	<0·0001	3·0 (0·0–81·9)	<0·0001
Female	506	72·8 (12·6–155·7)		18·1 (0·0–47·7)		3·5 (0·0–76·7)		25·8 (2·4–62·9)		4·6 (0·0–67·4)	
Age Group											
<2	74	76·5 (39·5–129·5)	0·001	18·0 (7·5–34·1)	0·02	3·6 (0·0–65·4)	0·6	26·7 (9·0–54·1)	0·2	2·8 (0·0–52·5)	<0·0001
2–9	865	77·1 (3·0–162·4)		15·6 (0·0–97·0)		4·8 (0·0–84·2)		23·4 (2·4–62·9)		3·4 (0·0–76·1)	
10+	143	68·5 (24·2–134·2)		17·5 (1·1–47·7)		6·3 (0·0–72·3)		25·2 (0·8–62·9)		8·4 (0·0–81·9)	
WBC											
<10	552	76·5 (12·6–162·4)	0·4	15·8 (0·0–62·2)	0·7	5·0 (0·0–84·2)	0·7	24·0 (0·8–57·4)	0·5	4·4 (0·0–67·4)	0·03
10‐	186	76·7 (24·2–136·7)		15·9 (0·0–40·4)		4·6 (0·0–68·5)		23·4 (2·4–62·9)		2·9 (0·0–76·1)	
20‐	179	76·9 (39·3–112·8)		16·9 (2·8–45·7)		5·5 (0·0–65·4)		24·4 (4·0–50·6)		3·4 (0·0–81·9)	
50‐	84	75·3 (25·7–108·7)		15·9 (2·7–38·5)		2·4 (0·0–55·2)		22·8 (3·4–55·7)		4·6 (0·0–57·3)	
100‐	81	77·5 (3·0–105·0)		15·6 (3·4–97·0)		6·5 (0·0–45·8)		21·9 (6·4–62·9)		3·3 (0·0–53·4)	
Steroid											
Pred	586	77·2 (19·5–162·4)	0·2	15·2 (0·0–47·7)	0·005	5·0 (0·0–84·2)	0·9	23·6 (0·8–58·2)	0·5	3·4 (0·0–81·9)	0·02
Dex	496	76·1 (3·0–155·7)		16·7 (0·0–97·0)		4·8 (0·0–76·7)		23·7 (2·4 –62·9)		4·3 (0·0–73·8)	

Values are given as median (range). The age groups are given as age in years at disease diagnosis. WBC = white blood cell count (x10^9^/l) at disease diagnosis. Pred = randomized and non‐randomized to prednisone. Dex = randomized and non‐randomized to dexamethasone.

**Table 3:** Differences in cytopenias between the ALL97 and ALL97/99 trial phases

Patients taking MP or TG only	ALL97		ALL97/99		Both phases	
	n	Median	n	Median	n	Median
TG, % weeks with thrombocytopenia (<100 × 10^9^/l)	202	7·7 (0·0–63·3)	31	4·5 (0·0–57·2)	233	6·9 (0·0–63·3)
MP, % weeks with thrombocytopenia (<100 × 10^9^/l)	245	3·4 (0·0–57·0)	336	2·2 (0·0–81·9)	581	2·4 (0·0–81·9)
Difference		3·5		1·1		3·5
95% CI		2·3–4·7		0·0–3·1		2·4–4·5
*P* value		<0·0001		0·06		<0·0001
TG, % weeks with neutropenia (<1·0 × 10^9^/l)	202	21·7 (2·4–53·2)	31	23·4 (9·2–48·8)	233	22·2 (2·4–53·2)
MP, % weeks with neutropenia (<1·0 × 10^9^/l)	245	25·9 (3·5–57·2)	336	24·9 (0·8–62·9)	581	25·4 (0·8–62·9)
Difference		−3·8		−1·4		−2·9
95% CI		−5·7 to −1·9		−4·8–2·3		−4·4 to −1·4
*P* value		0·0001		0·5		0·002
TG, % weeks with neutropenia (<0·5 × 10^9^/l)	202	9·7 (0·0–31·2)	31	9·0 (2·0–26·0)	233	9·5 (0·0–31·2)
MP, % weeks with neutropenia (<0·5 × 10^9^/l)	245	11·3 (0·0–35·1)	336	10·0 (0·0–31·8)	581	10·2 (0·0–35·1)
Difference		−1·6		−1·2		−1·1
95% CI		−2·7 to −0·6		−3·3–0·8		−2·0 to −0·3
*P* value		0·002		0·2		0·009

The above analysis includes only those patients taking randomized mercaptopurine (MP) or thioguanine (TG) throughout maintenance. Values are given as median (range). *n *= number of patients in each group. CI = confidence interval.

There was no difference in the % time with a neutrophil count <0·5 × 10^9^/l between the trials (Table 2). There was no difference in % time with neutropenia between thioguanine or mercaptopurine within ALL97/99 (Table 3). Within ALL97, those taking mercaptopurine experienced more neutropenia than those taking thioguanine whilst those taking thioguanine experienced more thrombocytopenia (Table 3). This increased myelosuppression was reflected by an increased duration of drug withdrawal and increased time spent on escalated doses in ALL97 (Table 2) and is a direct reflection of the differing dose titration protocols between the ALL97 and ALL97/99 phases of the trial; the former was more aggressive, adjusting the dose monthly if cell counts remained above threshold whist the latter aimed for controlled myelosuppression with dose adjustment at the start of a 3‐month maintenance cycle to keep cell counts within a therapeutic window.

Despite the difference in dose titration protocols between ALL97 and ALL97/99, throughout both phases of the trial girls experienced more myelosuppression, both episodes of neutropenia and thrombocytopenia, than boys (ALL97 neutropenia <0·5 × 10^−9^/l, girls 25·6% of time, boys 21·6%, *P *< 0·0001; ALL97/99 girls 25·8%, boys 22·1%, *P *< 0·0004. ALL97 thrombocytopenia girls 5·7% of time, boys 4·3%, *P *= 0·02; ALL97/99 girls 3·5%, boys 2·1%, *P *= 0·002). The increased myelosuppression experienced by the girls was reflected in thiopurine dose intensity, girls had significantly less time at escalated doses, experienced more dose withdrawal and received a lower average thiopurine dose (Table 2). The average daily dose was lower in girls in both phases of the trial (ALL97 girls 73·6%, boys 80·5%, *P *< 0·001; ALL97/99 girls 71·0%, boys 78·8%, *P *< 0·0001). There was no heterogeneity with respect to randomized thiopurine or *TPMT* genotype.

### Thiopurine dosage and TPMT genotype

Dose intensity data was available for 818 of the 1334 (61%) children with *TPMT* genotypes. There was a significant heterogeneity in dose tolerance by genotype (Table 4). Only two children homozygous for *TPMT* variant alleles (TPMT deficiency) had full dose intensity data available: both of these girls were randomized to mercaptopurine; mercaptopurine tolerance differed between the two. Due to more myelosuppression the TPMT*2/*3A compound heterozygote tolerated an average daily dose of 12·6% (9·45 mg/m^2^) and the TPMT*3C/*3C homozygous mutant 19·5% (14·6 mg/m^2^). Comparing the *TPMT* wild‐type (TPMT*1/*1) patients with the *TPMT* wild‐type/variant allele heterozygotes, the former had a higher average dose than the latter (78% versus 70% respectively, *P *< 0·0002) and experienced less time with the dose withdrawn due to cytopenias (15·5% vs. 20·8% of time respectively, *P *< 0·001).

**Table 4:** Thiopurine dosage and myelosuppression by *TPMT* genotype

TPMT subgroup	*TPMT* genotype	n	Average dose (% of protocol)	*P*	% time at no dose	*P*	% time dose escalated	*P*	% time with neutropenia (<0·5 × 10^9^/l)	*P*	% time with thrombocytopenia (<100 × 10^9^/l)	*P*
All genotypes	*1/*1	735	78·0 (29·8–162·4)	0·001	15·5 (0·0–62·2)	0·0009	5·8 (0·0–84·2)	0·05	23·4 (0·8–62·9)	0·1	3·4 (0·0–73·8)	<0·0001
	*1/*3A	66	70·1 (24·2–155·7)		21·3 (5·1–46·8)		1·5 (0·0–76·7)		26·2 (9·7–57·4)		8·4 (0·0–76·1)	
	*1/*3C	9	72·5 (3·0–83·1)		25·0 (10·2–97·0)		2·4 (0·0–4·9)		22·4 (13·5–37·3)		8·8 (1·5–57·0)	
	*1/*2	2	92·8 (82·8–102·7)		19·3 (15·8–22·9)		33·3 (19·3–47·4)		18·4 (16·9–20·0)		14·1 (13·7–14·5)	
	*1/*21	1	57·4		25·0		0·0		39·3		15·5	
	*1/*9	1	82·1		8·6		2·1		25·0		0·7	
	*2/*3A	1	12·6		34·6		0·0		37·0		46·9	
	*3C/*3C	1	19·5		14·1		0·0		15·3		2·4	
	*1/*33	1	61·8		11·9		10·7		20·2		1·2	
	*1/*34	1	61·9		19·7		2·6		34·2		14·5	
Group 1	*1/*1 vs	735	78·0 (29·8–162·4)	0·0002	15·5 (0·0–62·2)	<0·0001	5·8 (0·0–84·2)	0·007	23·4 (0·8–62·9)	0·02	3·4 (0·0–73·8)	<0·0001
	Other #	81	70·4 (3·0–155·7)		20·8 (5·1–97·0)		2·4 (0·0–76·7)		25·3 (9·7–57·4)		8·8 (0·0–76·1)	
Group 2	*1/*1 vs	735	78·0 (29·8–162·4)	0·0009	15·5 (0·0–62·2)	<0·0001	5·8 (0·0–84·2)	0·01	23·4 (0·8–62·9)	0·009	3·4 (0·0–73·8)	<0·0001
	*1/*3A	66	70·1 (24·2–155·7)		21·3 (5·1–46·8)		1·5 0·0–76·7)		26·2 (9·7–57·4)		8·4 (0·0–76·1)	
Group 3	*1/*3A vs	66	70·1 (24·2–155·7)	0·6	21·3 (5·1–46·8)	0·4	1·5 (0·0–76·7)	0·9	26·2 (9·7–57·4)	0·3	8·4 (0·0–76·1)	0·3
	*1/*3C	9	72·5 (3·0–83·1)		25·0 (10·2–97·0)		2·4 (0·0–4·9)		22·4 (13·5–37·3)		8·8 (1·5–57·0)	
Group 4	*1/*1 vs	735	78·0 (29·8–162·4)	0·04	15·5 (0·0–62·2)	0·05	5·8 (0·0–84·2)	0·1	23·4 (0·8–62·9)	0·9	3·4 (0·0–73.)	0·008
	*1/*3C	9	72·5 (3·0–83·1)		25·0 (10·2–97·0)		2·4 (0·0–4·9)		22·4 (13·5–37·3)		8·8 (1·5–57·0)	

Values are given as median and (range) when applicable. The *TPMT* homozygous variant genotype data was excluded from the *TPMT* subgroup, Group 1 to 4 comparisons. Other # = TPMT *1/*3A, *1/*3C, *1/*2, *1/*21, *1/*9, *1/*33, *1/*34. Vs = versus*. n *= number of patients in each group.

### Clinical outcome

For the complete trial [*n* = 1948; 5‐year EFS = 80%, overall survival = 89% (Vora *et al*, 2006)], there were significant differences by trial part, age group, presenting WBC and steroid, but no difference by gender or randomized thiopurine (Vora *et al*, 2006; Mitchell *et al*, 2009). In the subset of patients used in the dosing analysis (*n* = 1082) the effects of these covariates on overall EFS were similar to the complete trial.

#### Thiopurine metabolites

Twenty patients taking mercaptopurine (approximately 3% of the mercaptopurine cohort) had overt drug non‐compliance problems (nil metabolites) at some point during maintenance chemotherapy. Partial non‐compliance problems (<Q1 both metabolites for the *n* = 707 mercaptopurine cohort = <276 pmol TGNs and <4698 pmol MeMPNs) were seen in 10% of patients taking mercaptopurine. Twenty‐nine children taking thioguanine (6% of thioguanine patients) had TGNs <750 pmol/8 × 10^8^ red cells and were considered to have problems with compliance, 12 of these patients (approximately 3% of thioguanine cohort) had TGNs <500 pmol/8 × 10^8^ red cells (Lennard *et al*, 2013). After stratifying for trial, age group, sex, WBC and steroid used, patients accumulating <750 pmol TGNs/8 × 10^8^ red cells on thioguanine had worse EFS than compliant patients (odds ratio [OR] = 2·58, 95% CI: 1·11–5·7, *P *= 0·04); the difference was more marked (OR = 3·60, 95% CI: 1·34–9·69, *P *= 0·02) when the definition of non‐compliance was restricted to <500 pmol TGNs/8 × 10^8^ red cells. However, there was no difference in EFS for non‐compliant mercaptopurine patients for any endpoint examined. For all of the patients with trial metabolite data there was no relationship between thioguanine‐derived TGNs, mercaptopurine‐derived TGNs or mercaptopurine‐derived MeMPNs and EFS (which includes all patients from the start of treatment) or jEFS (which excludes all patients with events in year 1), when the thiopurine metabolites were analysed as either continuous variables or split into quartiles. The results for thiopurine metabolites remained unchanged when stratified by *TPMT* genotype, trial, age group, sex, WBC or steroid used.

#### Thiopurine dosage

Quartile analysis of the proportion of time spent on escalated doses showed that those patients who spent more time on escalated doses had a worse outcome (jEFS *P *= 0·04). There was no heterogeneity of effect of time spent on escalated doses within subgroups defined by genotype, randomized thiopurine or thiopurine taken. Quartile analysis showed no significant trend in jEFS for average % dose thiopurine taken or the proportion of time spent at reduced or no dose. Likewise, there was no significant difference in outcome seen by proportion of time spent with neutropenia or thrombocytopenia. There was no difference in jEFS between those patients who did not experience any episodes of thiopurine‐induced cytopenia and the majority of patients that did. There was no evidence of heterogeneity in sub‐groups defined by *TPMT* genotype.

#### *TPMT* genotype

**Figure 2:**
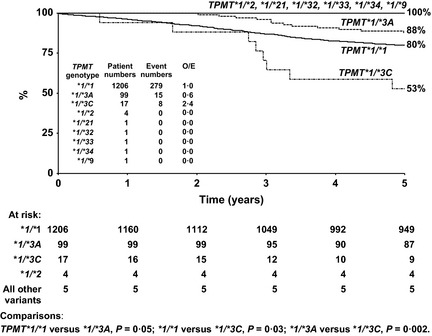
Event‐free survival by *TPMT* genotype. ALL97 and ALL97/99.

EFS differed significantly by *TPMT* genotype (Fig 2). Heterozygous TPMT*1/*3C patients fared worse (5‐year EFS, 53%, 95%CI 29–77%) compared to the other heterozygous *TPMT* patients (TPMT*1/*3A, *1/*2, *1/*21, *1/*9, *1/*32, *1/*33, *1/*34; EFS 89%, 95%CI 83–95%, *P *= 0·002) and homozygous TPMT*1/*1 patients (EFS 80%%, 95%CI 78–82%, *P *= 0·03). TPMT*1/*3A patients (EFS 88%, 95%CI 81–94%) fared significantly better than TPMT*1/*1 (EFS 80%, 95%CI 78–82%) patients (Fig 2). These differences remained after stratifying for ethnic origin. However, numbers of non‐white Caucasian children were small, specifically for the TPMT*1/*3A cohort (*n* = 99), 1 was Asian and 1 mixed race, and for the TPMT*1/*3C cohort (*n* = 17), 1 was Asian, 1 black and 2 mixed race. None of the TPMT*1/*3C children had CNS disease at diagnosis or Down syndrome, two had T‐cell immunophenotype and one of these relapsed. Seven TPMT*1/*3C patients were treated on ALL97 (5 girls, 2 boys) and 10 on ALL97/99 (5 girls, 5 boys). The median age of the TPMT*1/*3C children (3 years) was similar to the TPMT*1/*3A children (4 years).

There was no significant difference between the TPMT*1/*3C and TPMT*1/*3A patients with respect to mean daily dose or incidence of cytopenias, although the number of TPMT*1/*3C patients with full dose intensity data available was small (*n* = 9), (Table 4). One TPMT*1/*3C patient had difficulty tolerating mercaptopurine (average daily mercaptopurine dose 3%, 10% of time with drug withdrawn), DNA sequencing and measurement of the formation of MeMPN metabolites showed that this boy was not TPMT deficient. However, the TPMT activity (5·6 units/ml packed red cells) was at the extreme lower end of the *TPMT* heterozygous distribution (Lennard *et al*, 2013). Despite experiencing thrombocytopenia for 25% of the time (14% with neutropenia also) and drug withdrawal/reduction due to other mercaptopurine ‘sensitivities’, this child (treated on ALL97/99 regimen B) remains in remission.

In a multivariate Cox regression analysis, the worse survival for TPMT*1/*3C against TPMT*1/*3A (TPMT*1/*3C hazard ratio = 4·5, 95% CI 1·7–11·8, *P *= 0·003) and for TPMT*1/*3C against TPMT*1/*1 and all other *TPMT* variant heterozygous genotypes (Table 5) retained significance in models for overall EFS that also included the covariates trial, age group, WBC at diagnosis and steroid randomization.

**Table 5:** Multivariate Cox regression analysis of overall event‐free survival for *TPMT* genotype and significant covariates

	Hazard ratio	95% CI	*P*
*TPMT* genotype			
*1/*1	1·0		0·003
*1/*3A, *1/*2, *1/*21, *1/*9, *1/*32, *1/*33, *1/*34	0·6	0·4–1·1	
*1/*3C	3·2	1·5–6·8	
Trial part			
ALL97	1·0		0·02
ALL97/99	0·7	0·6–1·0	
Age (per year increase)	1·1	1·0–1·1	<0·0001
Log (WBC) (per unit increase)	1·1	1·0–1·2	0·04
Randomized steroid			
Pred	1·0		0·002
Dex	0·7	0·5–0·9	

Multivariate Cox regression analysis of overall event‐free survival defined from the start of treatment. Genotype comparison TPMT *1/*1 vs. (TPMT *1/*3A, *1/*2, *1/*21, *1/*9, *1/*32, *1/*33, *1/*34) versus TPMT*1/*3C, (*n* = 1189 patients, with *n* = 251 events). *TPMT* = thiopurine methyltransferase gene, CI = confidence interval, WBC = white blood cell count at diagnosis, Pred = prednisone, Dex = dexamethasone.

#### Second malignancies

There were 13 second cancers (8 ALL97, 5 ALL97/99) in 1935 trial patients (0·7%) occurring a median of 4·6 years (range 1·0 to 11·3) from the start of chemotherapy in children aged from 3 to 12 years (median 7) at diagnosis. Of these 13 patients, 12 had *TPMT* genotypes (11 TPMT*1/*1; 1 *TPMT* *1/*3A) and 11 had genotype and metabolite data and 7 had dosing data. One child (*TPMT* wild‐type), aged 4 years at diagnosis, was diagnosed with a brain tumour (grade 2 astrocytoma) 10·3 years after diagnosis. This child had CNS disease at ALL diagnosis and received cranial radiotherapy. There was no association between second cancer and genotype, thiopurine metabolites, thiopurine average % dose or frequency of cytopenias. There was no association between second cancer and type of thiopurine: although a higher proportion of second cancers occurred in those randomized to thioguanine, this difference was not significant (*P *= 0·41).

## Discussion

This study focused on the complex relationships between *TPMT* genotype, thiopurine dose intensity and myelosuppression, and the effect of these variables on treatment outcome within the ALL97 trials. Within the MRC UKALL X trial, a study of mercaptopurine dose intensity showed that those children who had one or more episodes of neutropenia (cell counts <0·5 × 10^9^/l and dose withdrawal) had a better prognosis than those who never became neutropenic (Chessells *et al*, 1997). Moreover, within UKALL X, those children randomized to receive intensive blocks were prescribed lower doses of mercaptopurine and became neutropenic more readily; the intensive blocks influenced the subsequent response to mercaptopurine maintenance chemotherapy. A reverse association of neutropenia with worse EFS was seen in a later U.S. study in which all children received intensive blocks of treatment. In a multivariate analysis, a higher dose intensity of mercaptopurine was a significant predictor of improved EFS. The worse EFS observed in those with a lower mercaptopurine dose intensity was due to neutropenia and the subsequent weeks of missed therapy due to low cell counts, rather than lower doses of mercaptopurine (Relling *et al*, 1999b).

In the current study, in which all children received intensive blocks, the thiopurine dose intensity (taken either as the % average daily dose prescribed or the time spent on reduced doses or dose withdrawal due to low cell counts) was not associated with treatment outcome. The duration of, or episodes of, neutropenia or thrombocytopenia were not associated with outcome.

The gender difference in dose tolerance and myelosuppression, initially reported in UKALL X (Chessells *et al*, 1997), remains within ALL97. However, unlike UKALLX, there is no gender difference in outcome. The latter is perhaps a reflection of the extra year of exposure to thiopurines experienced by the majority of boys. TGN concentrations, metabolite concentrations associated with thiopurine‐induced myelosuppression, differ with respect to *TPMT* status in both boys and girls. For both genders and both thiopurines, *TPMT* heterozygous patients accumulated significantly higher TGN concentrations compared to the *TPMT* wild‐type cohort (Relling *et al*, 1999a; Lennard *et al*, 2013). The *TPMT* heterozygotes tolerated a significantly lower average daily thiopurine dose than the *TPMT* wild‐type patients and experienced more cytopenias. This is in keeping with recent studies within Berlin‐Frankfürt‐Münster (BFM) protocols that have reported an increased sensitivity of patients with *TPMT* heterozygosity to mercaptopurine‐induced myelosuppression (Karas‐Kuzelicki *et al*, 2009; Peregud‐Pogorzelski *et al*, 2011).

There is some debate as to whether the *TPMT* heterozygote should be prescribed a lower thiopurine dose than the *TPMT* wild‐type patient; not all *TPMT* heterozygotes are intolerant of thiopurine. The current general advice is a lower starting dose for the *TPMT* heterozygote with a titration‐upwards approach to an acceptable degree of myelosuppression (Relling *et al*, 2011, 2013). In the present study, however, the *TPMT* heterozygotes tolerated significantly lower average % dosages than the *TPMT* wild‐type patients (70% vs 78% for *TPMT* wild‐type, a daily‐dose difference of 6 mg/m^2^ per day mercaptopurine or 3·2 mg/m^2^ per day thioguanine). However, the range of thiopurine dosages tolerated was wide, with the upper and lower limits similar for both *TPMT* genotypes. These findings do not support any change in the prescribing criteria (both genotypes start at the same standard protocol dose and titrate to toxicity) for the UK ALL trials with respect to the *TPMT* heterozygous patient; a similar conclusion to that reached with respect to mercaptopurine dosages in the German BFM protocols (Stanulla *et al*, 2005).

TPMT *1/*3A patients had a better EFS than TPMT *1/*1 patients, the former also experienced more cytopenias and accumulated higher TGN concentrations than the latter. However, neither the reference TGN concentration nor the frequency of cytopenias throughout thiopurine chemotherapy were directly associated with EFS. Survival was inexplicably worse for patients with TPMT*1/*3C than for TPMT*1/*3A patients. Despite similar mercaptopurine dosages and TPMT activities, the TPMT*1/*3C patients accumulated significantly less TGNs and lower MeMPN concentrations than TPMT*1/*3A patients; this could indicate an increased frequency of non‐adherence and suboptimal metabolite exposure in the TPMT*1/*3C cohort. The differences in survival for the TPMT*1/*3C with respect to TPMT*1/*3A patients could explain why, in a previous single nucleotide polymorphism analysis in a smaller cohort of ALL97 patients (Matimba *et al*, 2014), we found no difference in EFS between *TPMT* genotypes when analysed as wild‐type or variant allele.

Compliance with oral chemotherapy was a confounding factor in this study. Although low or absent metabolite concentrations may be due to other factors, non‐compliance with oral chemotherapy is the most likely cause in this group of patients. It is possible that some parents may not have followed the clear guidance on evening dosing and avoiding food and milk products within 1 h of the thiopurine dose, and some patients may have had poor absorption due to other causes. However, in such cases, the apparent drug dose is reduced but the inverse relationship between TGN and MeMPN metabolite formation should be maintained. This is in contrast to the very low levels of both metabolites seen in non‐compliance. There are few studies on non‐compliance with oral anticancer therapy in children or adolescents, most studies focus on adults (Verbrugghe *et al*, 2013). Assessment of mercaptopurine non‐compliance in previous UK ALL trials, by structured interview, associated low metabolite concentrations at high drug doses with admitted failure to take the tablets (Davies *et al*, 1993). A similar evaluation of non‐compliance in ALL children by clinical (structured interviews and evaluation of medical charts) and laboratory (mercaptopurine metabolite monitoring) indices associated lower non‐compliance with adverse socioeconomic factors (De Oliveira *et al*, 2004). Non‐compliance perhaps explains the worse outcome for those who spent a longer time on escalated thiopurine dosages; an effect independent of *TPMT* status. Non‐compliance with thioguanine therapy, as previously defined by low TGN levels (Lennard *et al*, 2013), was also associated with a worse EFS whilst non‐compliance with mercaptopurine was not. This is perhaps explained by action taken by the clinician to address the non‐compliance identified by the metabolite results in the case of mercaptopurine but not thioguanine, because thioguanine‐derived TGN levels suggestive of non‐compliance were derived after the trial closed (Lennard *et al*, 2013). If unaddressed, a lower compliance to mercaptopurine is known to increase the relapse risk, and ethnic differences in relapse risk have been associated with increased non‐adherence (Bhatia *et al*, 2012).

*TPMT* heterozygotes taking mercaptopurine were found to have a lower relapse risk in the Nordic Society for Paediatric Haematology and Oncology (NOPHO) ALL‐92 trial (Schmiegelow *et al*, 2009a) but the benefit was offset by a higher incidence of second cancers (Schmiegelow *et al*, 2009b). With a median follow‐up of 11·3 years, we have not observed an excess of second cancers in *TPMT* heterozygotes as reported in the NOPHO and St Jude total therapy trials; the latter studies linking lower TPMT activity with second brain tumours in children who received radiotherapy and with etoposide mediated myeloid leukaemias (Relling *et al*, 1998, 1999c; Schmiegelow *et al*, 2009b). Neither NOPHO nor ALL97 include etoposide. In the NOPHO and St Jude trials, high‐dose methotrexate and cranial irradiation are given alongside oral thiopurines, the former is not included in the ALL97 trials whilst the latter was reserved for patients (<5%) with overt CNS disease at diagnosis (Vora *et al*, 2006). The BFM studies report that *TPMT* status is not a risk factor for the development of second cancers (Stanulla *et al*, 2009). In the BFM protocols, some of which contained cranial irradiation, mercaptopurine was given alongside high‐dose methotrexate but at much lower dosages (25 mg/m^2^ per day) than the NOPHO and St Jude trials (75 mg/m^2^). The higher doses of mercaptopurine in the latter trials, alongside cranial irradiation and/or high‐dose methotrexate, could have contributed to the development of second malignancies. A subsequent NOPHO trial (NOPHO ALL‐2000) reported that using reduced mercaptopurine dosages for the *TPMT* heterozygote patient, alongside high‐dose methotrexate, reduced the risk of developing second cancers but this was counterbalanced by an increased risk of relapse for the *TPMT* heterozygote (Levinsen *et al*, 2014).

The data reported here will allow a more informed use of thiopurine drugs. Within the UK ALL trials, thiopurine‐induced cytopenias did not have a detrimental effect on EFS. A reference TGN metabolite concentration, taken at an early stage in thiopurine therapy when the patient is tolerating the drug, is useful for subsequent comparisons of metabolite exposure but is, in itself, not predictive of EFS. Some patients exhibited variable compliance with their oral thiopurine therapy, which was shown to have a negative effect on EFS for thioguanine patients. Other than possible increased non‐compliance problems in TPMT *1/*3C patients, a cohort with a higher proportion of ethnic minorities, the worse survival for the TPMT *1/*3C patients is unexplained. The worse outcome for TPMT *1/*3C will be re‐examined within the UK ALL 2003 trial, the successor to ALL97, in which treatment intensity was adjusted based on minimal residual disease risk stratification. The EFS for ALL2003 (5‐year EFS for low‐risk patients >94%) (Vora *et al*, 2013) is far superior to the ALL97 trials and this may negate any impact of *TPMT*.

## Authorship

LL, RW, AV contributed to the design of the study. AV was a trial co‐ordinator. LL, CSC conducted the biochemical measurements. LL, CSC, RW were involved in data collection and data analysis. LL, CSC, RW, AV were involved in data interpretation. LL wrote the manuscript. All authors were involved in the revision and editing of the manuscript. All authors approved the final version of the manuscript.

## Competing interests

The authors have no competing interests.
